# Power-duration relationship comparison in competition sprint cyclists from 1-s to 20-min. Sprint performance is more than just peak power

**DOI:** 10.1371/journal.pone.0280658

**Published:** 2023-05-26

**Authors:** Hamish Ferguson, Chris Harnish, Sebastian Klich, Kamil Michalik, Anna Katharina Dunst, Tony Zhou, J. Geoffrey Chase

**Affiliations:** 1 Centre for Bioengineering, Department of Mechanical Engineering, University of Canterbury, Christchurch, New Zealand; 2 Department of Exercise Science, Murphy Deming College of Health Sciences, Mary Baldwin University, Fishersville, Virginia, United States of America; 3 Department of Paralympic Sport, Faculty of Physical Education and Sports, Wroclaw University of Health and Sport Sciences, Wroclaw, Poland; 4 Institute of Applied Training Science, University of Leipzig, Leipzig, Germany; Universidad de Castilla-La Mancha, SPAIN

## Abstract

Current convention place peak power as the main determinant of sprint cycling performance. This study challenges that notion and compares two common durations of sprint cycling performance with not only peak power, but power out to 20-min. There is also a belief where maximal efforts of longer durations will be detrimental to sprint cycling performance. 56 data sets from 27 cyclists (21 male, 6 female) provided maximal power for durations from 1-s to 20-min. Peak power values are compared to assess the strength of correlation (R^2^), and any relationship (slope) across every level. R^2^ between 15-s– 30-s power and durations from 1-s to 20-min remained high (R^2^ ≥ 0.83). Despite current assumptions around 1-s power, our data shows this relationship is stronger around competition durations, and 1-s power also still shared strong relationships with longer durations out to 20-min. Slopes for relationships at shorter durations were closer to a 1:1 relationship than longer durations, but closer to long-duration slopes than to a 1:1 line. The present analyses contradicts both well-accepted hypotheses that peak power is the main driver of sprint cycling performance and that maximal efforts of longer durations out to 20-min will hinder sprint cycling. This study shows the importance and potential of training durations from 1-s to 20-min over a preparation period to improve competition sprint cycling performance.

## Introduction

Sprint track cycling at Olympic Games and Elite World Championship levels is a subsection of cycle sport requiring a combination of peak speed, power, strength and short term endurance (speed endurance) [1]. While sprint cycling requires both a high level of strength and power, the nature of racing is actually more within the speed-endurance realm [2]. Unlike Olympic Weightlifting, powerlifting, or peak power field events, all sprint cycling races (team sprint, 500/1000-m time trial, match sprint and Keirin) range in durations from 15-s to 75-s depending on the event [1, 3]. Events longer as 75 s duration are performed by endurance cyclists specialized in pursuit races (team and individual), omnium and madison taking between 135–300 seconds [4–6]. More recent study has focused on the selection of a gear to rapidly achieve a maximum cadence to manage fatigue and sustain sprint performance over race durations [7–9].

The principle of specificity is a key component of preparation for sprinting performance [10–12]. Current models of sprint cycling performance appear inadequate to develop optimal performance in competition sprint cycling [1]. The reliance on peak power as a singular, simple, and main predictor of sprint cycling performance appears to be more about convenience than empirical evidence [13–16]. In particular, ergometer-based testing performed in the laboratory is widely used to assess this value, while successful performance in track-sprint cycling is multi-factorial, incorporating physiological traits, tactics, and other specific skills [17–21].

Despite data showing the reliance on both glycolytic and oxidative energetic pathways for durations associated with competition sprint cycling [22–26], the priority of training is focused on peak power [27], peak strength in the gymnasium [14, 16], and maximal torque after the cycling-specific isometric resistance training [28]. The application of this approach has limited empirical evidence [3, 29]. Stone et al. [29], show a relationship between various strength exercise measures and standing start performance (25-m– 333.33-m). However, this research lacks a comparison with longer durations to determine if the relationship remains as distance increases. Dorel et al. [3], concluded, peak power relative to frontal area or pedalling frequency, was a strong predictor of flying 200-m performance on a velodrome, however the study did not investigate the role of average power, average cadence or average frontal area/power on flying 200-m performance.

The anaerobic speed reserve, developed to model track running performance for events from 10-s– 300-s, is based on maximal sprinting speed and maximal aerobic speed (the lowest running velocity at which $\dot{V}$O_2MAX_ may occur) [30]. Using a power meter, this concept has been applied to cycling, called the anaerobic power reserve (APR) [31]. Similarly, the critical power (CP) model has been used to model performance over short, medium and long durations reflecting exercise domains (heavy, severe, extreme) [32–34]. An advantage of this model is it allows the determination of a critical power for durations between 2–15 minutes [35], and also an estimate of finite high intensity energy referred to as W’ and measured in kilojoules [32].

The APR and CP models may provide a useful means of predicting the power-duration relationship in the extreme exercise intensity domain, such as during sprint track cycling events Leo et al. [35]. It is questionable whether it is necessary to run multiple trials to predict performance given the ease of measuring actual performance [36], modelling the determinants from actual performance [7, 37], and measuring performance with various sensors, especially power output, during competition [38].

While the literature is replete with studies on peak power in relation to sprinting, some coverage of performance over competition durations of 15-75s, and sparse reporting on multiple sprint performance (qualifying rounds, intermediate rounds, and medal finals) which form the basis of all world championship sprint events, there is no research on the effect of longer duration efforts within a training period on the performance of sprint cyclists. This study compares mean maximal power in sprint cycling across a wider range of durations from 1-s to 20-min to assess the validity of assumptions in current training approaches [14, 16], to show inter-relationships of power across a range of training durations in sprint track cyclists. We hypothesised:

1. There are strong consistent correlations between sprint cycling power at two common sprint competition durations of (15-s and 30-s) to power durations from 1-s to 20-min.
2. The slope of the regression indices will show all durations measured from 1-s to 20-min have a positive effect on sprint cycling performance.

## Methods

### Data acquisition

Data were obtained from the publicly available open-access fitness depository www.strava.com [39], and downloaded according to Strava Privacy Policy (https://www.strava.com/legal/privacy) with no identification of people used in the study. Based on the public accessibility of the data the University of Canterbury Human Research Ethics Committee exempted this research from seeking ethics approval (2022/06/EX) without need of informed consent. Sprint cyclists were identified by matching Strava data with performances at New Zealand national track cycling championships [40], and World Masters Track Cycling Championships [41]. We used data from Strava and Cycling NZ to determine weight, sex and age for the rider data. The Sauce extension (https://www.sauce.llc/) to download a.tcx format file containing power meter data from Strava data of athletes competing at New Zealand sprint cycling competitions. Ride files uploaded to Strava from competition, and training sessions for 3–12 months prior to each NZ sprint championship event with available data.

### Participants

29 participants that included 9 junior U17—age 15/16 yo in year of competition; 4 U19—age 17/18 yo in year of competition; 8 elite—age 19–34 yo in year of competition; and 8 masters—35+ yo with assignment in 5-year age groups (8 participants), sprint track cyclists (Table 1). The classification of sprint track cyclists was made from the ability to place top 4 in the sprint in U17, U19, and Elite levels, top 4 in Keirin for U19, Elite and Masters, top 4 in time trial (500-m for women, 1000-m for men) in all grades, competing at Junior World level in sprint events, and top 4 placing in Masters World Championship events. Data from power meter measures were collected during testing, training, and racing sessions over a cross-section of male [21] and female [6] track cyclists. Data from endurance track cyclists used in the authors’ prior research were used as a comparison group; power meter measures were collected during testing, training, and racing sessions over a cross-section of 21 male and female track cyclists [2].

**Table 1:** Participant data including this study, endurance (END) cyclists. Median and interquartile range (IQR).

**Data Sets**	56	44	12
**Participants**	27	21	6
**Age [years]**	19 [16–39]	25 [16–40]	17 [16–19]
**Weight [kg]**	76.5 [74.0–86.8]	80.3 [75.0–88.0]	64.0 [60.0–74.0]
**END Data Sets**	144	128	16
**END [Age]**	26 [17–38]	27 [17–39]	19 [17–30]
**END [Weight]**	70.0 [65.0–75.5]	72.0 [67.0–76.0]	57.0 [56.0–63.5]

### Study overview

We firstly distinguished data in this study against to group of endurance track cyclists to assure we were testing data from sprint cyclists [2]. The 15-s duration reflects the flying 200-m where the riders jump 100-m—125-m from the 200-m mark on the track, the first lap of Team Sprint and the shortest duration match sprints take place over. Similarly, 30-s duration reflects a 500-m time trial, riding *2nd wheel* (2nd rider in the starting line-up) in the team sprint, a long match sprint, and/or the Keirin event. These two common durations of sprint cycling where compared against several durations from 1-s to 20-min to test our two hypotheses.

### Power meter data

This study uses power meter data, which measures power in watts at 1-s intervals, as variables. We select cyclists who use a power meter model that allows regular calibration [42] and perform a zero offset before each use to account for temperature changes based on racing observation. 28 of the cyclists used an SRM (SRM, Jülich, Germany) brand power meter and one used an Infocrank (InfoCrank Classic, Verve Cycling, Perth AUS) brand. We upload this data to the WKO5 (TrainingPeaks, Boulder, CO) sport data analysis software. We obtain the max mean power for each participant for 1-s, 6-s, 12-s, 15-s, 18-s, 24-s, 30-s, 40-s, 50-s, 60-s, 90-s, 2-min, 3-min, 6-min, 12-min, and 20-min durations in WKO5.

We analysed this data to determine the relationships between all durations and two common sprint cycling performance durations (15-s and 30-s). We also assessed and plotted the slope for the two sprint durations and all other durations compared to a 1:1 relationship (15-s:15-s and 30-s:30-s power). The data included maximal efforts over all durations from racing, testing and training sessions. We used endurance cyclist data from previous research [2], to validate our data and compare with our sprint cyclist data.

### Analysis

We used Matlab version R2021a (The MathWorks, Natick, MA) to perform the descriptive statistics including mean maximal power and interquartile range and the statistical analysis. We assessed model quality by total least squares correlation coefficient, R2, to account for variability and error in both variables [43, 44]. A higher R^2^ value indicates a stronger relationship and a better model and predictor. Model slope indicates the trade-off between power interval training and peak 1-s power. We used Jamovi version 2.2.5 (The Jamovi Project, Sydney, Australia) statistical software to conduct a Welch’s t-test for unequal sample sizes and variances to compare the differences in power between the sprint and endurance groups to ensure the study group were sprint cyclists in terms of power metrics [45]. *P*-values of <0.05 for the *t*-test were considered statistically significant.

## Results

### Assuring a sprint cohort

**Fig 1:**
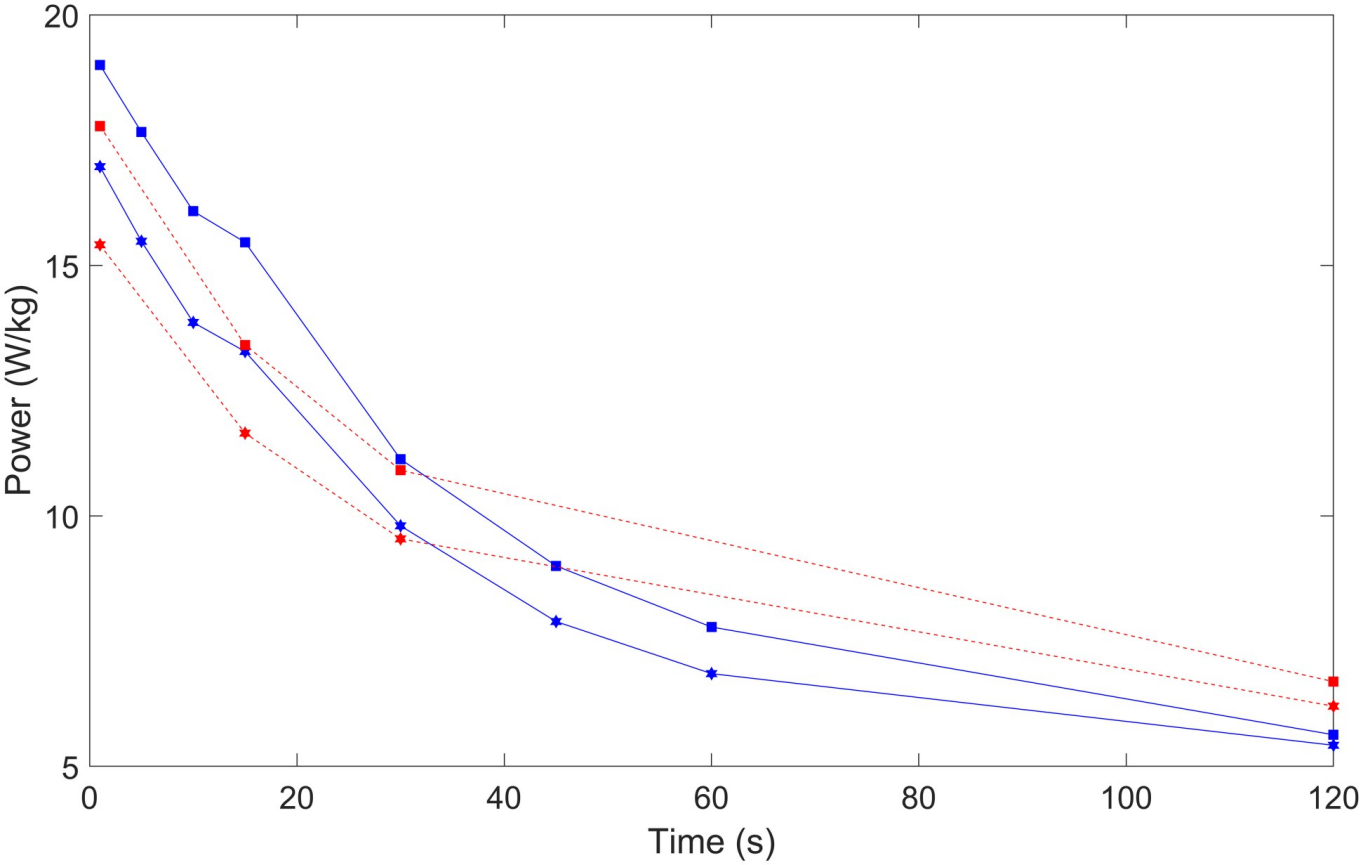
The comparison of power (W/kg) for female (hexagon) and male (square) sprint (blue line) and endurance (red dotted line) track cyclists from 1-s to 2-min measuring peak power.

Table 2 shows the results of the Welch’s *t*-test between sprint and endurance cyclists from prior research [2]. It describes the difference between sprint and endurance groups for all durations except 30-s W/kg. The sprint group delivers higher power than the endurance group for durations below 30-s and lower power for durations above 30-s (3-min and 20-min). These data indicate that the national level sprint cyclist data collected were different from a group of national level endurance cyclists in an expected way for sprint cyclists. Fig 1 illustrates the differences between the male and female sprint and endurance groups.

**Table 2:** Independent samples T-Test between sprint power output in watts per kilogram (PO) and endurance PO for selected durations.

	Statistic	df	*P*
**1-s PO**	2.362	73.1	0.021
**15-s PO**	2.163	70	0.034
**30-s PO**	0.612	74.4	0.542
**3-min PO**	-6.591	64.4	< .001
**20-min PO**	-10.447	68.5	< .001

### Sprint cycling power across durations

Table 3 shows the R^2^ values and slopes for 15-s and 30-s against power at all durations. The main result was the high coefficient of determination for 15-s and 30-s (R^2^ ≥ 0.86; R^2^ ≥ 0.83, respectively). In particular, correlations were very strong for durations longer than the 15-30-s durations most associated with sprint cycling performance.

**Table 3:** R^2^ and slopes for 15 and 30 second power, and power at all durations.

Time	R^2^ 15-s	Slope 15-s	R^2^ 30-s	Slope 30-s
**1-s**	0.94	0.78	0.89	0.59
**6-s**	0.97	0.83	0.90	0.65
**12-s**	0.99	0.92	0.91	0.73
**15-s**	**1.00**	**1.00**	0.93	0.78
**18-s**	0.99	1.07	0.94	0.82
**24-s**	0.96	1.16	0.98	0.93
**30-s**	0.93	1.26	**1.00**	**1.00**
**40-s**	0.90	1.50	0.97	1.16
**50-s**	0.89	1.65	0.94	1.30
**60-s**	0.89	1.84	0.93	1.41
**75-s**	0.88	2.09	0.91	1.58
**90-s**	0.88	2.23	0.90	1.72
**2-min**	0.88	2.47	0.89	1.92
**3-min**	0.87	2.67	0.87	2.09
**6-min**	0.87	3.25	0.85	2.47
**12-min**	0.86	3.59	0.84	2.76
**20-min**	0.86	3.88	0.83	2.96

**Fig 2:**
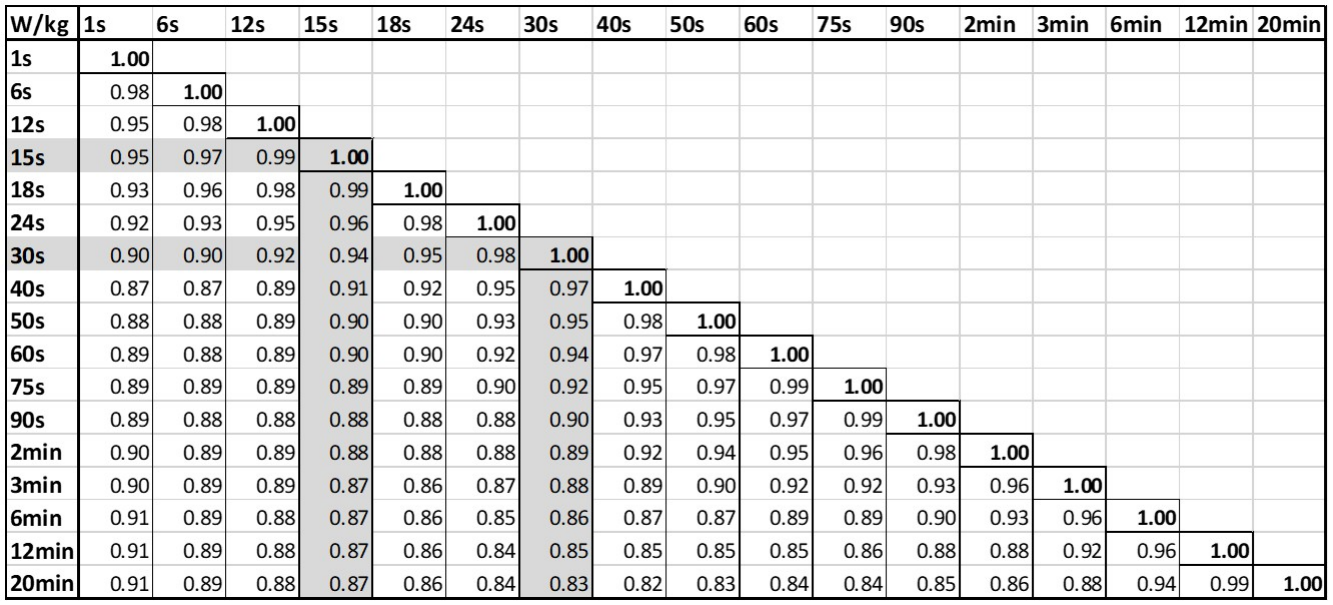
R^2^ comparing correlation across all durations. The values for sprint cycling durations of 15-s and 30-s are highlighted showing consistent strong correlation across all power ranges.

Similarly, Fig 2 shows the matrix of correlation coefficients comparing all power durations. Each power was perfectly correlated to itself. The minimum values are all R^2^ ≥ 0.83, showing there are no distinguishable or large differences in correlations between power across all durations [46]. Table 3 and Fig 2 respectively support our first hypothesis.

**Fig 3:**
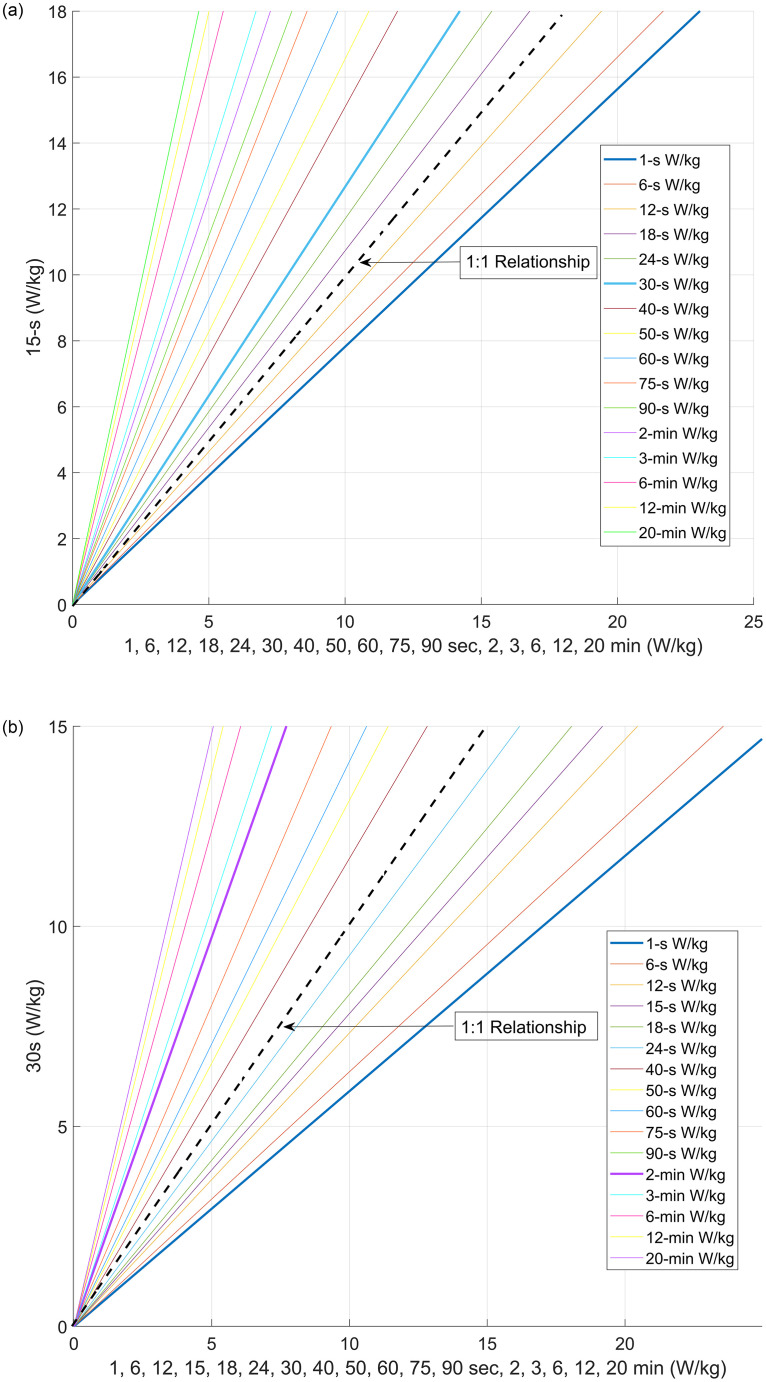
TOP: Lines for watts/kg 15-s against W/kg for all durations studied; and BOTTOM: Slope’s for W/kg 30-s against W/kg for all durations studied.

The slopes in Table 3, and Figs 3a and 3b show an increase in power at all durations has a positive effect on two common durations of sprint cycling performance. An increase in any max mean power from 1-s to 20-min had a positive effect on two common durations of sprint cycling performance. Fig 3 and Table 3 respectively confirm our second hypothesis.

## Discussion

The purpose of this study was to compare 15-s and 30-s power in sprint cyclists, with a range of durations from 1-s to 20-min. We hypothesised strong correlations across all ranges of power measured from 1-s to 20-min, contrary to current coaching belief and practice. Our data are the first to demonstrate strong relationships between two common durations of sprint cycling competition performance with power durations from 1-s to 20-min, therefore we propose that training durations within this range, is beneficial. Maximal efforts of up to 20-min may be warranted to complement sprint cycling training, and do not detract from sprint cycling performance within a training period of 3–12 months.

Slopes for all power durations indicate the strongest relationships are for 15-s and 30-s power (Fig 2). The 1-s power slope, has the same slope as 2-min power, which indicates all the durations between these values are important to train to influence sprint performance. This outcome is also contrary to current coaching practice and belief in the field, and these results provide significant evidence supporting a different approach.

In particular, 1-s power predicts performance for both 15-s and 30-s power, but 6-s, 12-s, 18-s, and 24-s power predict 15-s power better. Likewise, 6-s to 90-s power (except for 20-s and 30-s) predict 30-s power better than 1-s power. This suggests that longer time intervals should be used in power-duration modelling, not only the peak power in the ~10–15 s sprint effort, as Sanders and Heijboer [31] also proposed.

This analysis can help profile cyclists who need to race maximally for 15–30 s, such as in sprint and Keirin events. The exact duration of each race depends on the group dynamics [1, 20, 21]. Cyclists who have high power output but are above the line of best fit may need to increase their power endurance and repeatability. These are important for medal-winning in championship sprint events that require multiple efforts.

For example, a powerful rider who is far from the line may need to train for longer power duration. The data shows that 20-min effort relates to 15-s and 30-s sprint performance. These stimuli could change depending on the training phase and the key events or season. A rider below the line may need to train for shorter and more intense power. The further from the line, the shorter the training duration to increase their power for sprint cycling.

A limitation of this data is that the group consists of national level sprint cyclists in Junior (under 17), under 19, elite and masters categories, and riders in age group world events: Junior Worlds [16–18] and masters (35+ in 5-year groups). Our data differs from endurance cyclists’ data, but our participants may do more endurance riding and racing than a high-performance group. This difference may reflect tradition or analysis of sprint cycling performance demands.

This data is from national level sprint cyclists who trained and raced for 3–6 months. They likely did 20-min peaks early in a general preparation phase [47]. Shorter duration efforts are more likely to be performed in the lead up to competition. This commonly reflects the periodisation of training focusing on three distinct periods of general preparation, specific preparation and peaking for key events [47]. Some training programmes utilise a reverse periodisation approach referred to as *short to long*, focusing on peak power and speed endurance added at the end, leading into competition [48]. However, the data presented here suggests a focus on 1-s is potentially to the detriment of actual performance when developing power around competition times has a closer relationship.

Future research should develop power profiles for sprint cyclists to see if they need to build capacity or power for sprint cycling durations. Elite programmes should try both capacity and 1-s power. With strong links between sprint power and 20-min power, longer efforts may help develop better sprint cyclists.

## Conclusions

The study compared maximal efforts for peak, short, medium and long term power with power at 15-s and 30-s sprint cycling durations. The strongest links were with durations around 15-s and 30-s, confirming our first hypothesis. Performance at 2-min to 20-min durations correlated strongly, and did not harm 15-s or 30-s power, in fact enhanced it, confirming our second hypothesis. Coaches can use this data to plan training that use endurance durations to improve sprinting performance.

## Supporting information

S1 File (DOCX)

S2 File (XLSX)
