# Supplementary material for: Power-duration relationship comparison in competition sprint cyclists from 1-s to 20-min. Sprint performance is more than just peak power
Source: PLoS One. 2023 May 26;18(5):e0280658. doi: 10.1371/journal.pone.0280658 (PMC10218722; doi:10.1371/journal.pone.0280658)
Supplement: S1 File — (DOCX) [file pone.0280658.s001.docx]

x_data = W2m;

x_data_label = '2-min Watts';

y_data = W30s;

y_data_label = '30-s Watts';

male_results_only = 0;

female_results_only = 0; % if both are 1, code should default to male results

% Set up seperate data here if just using male or female data

% [Male_results_x, Male_results_y, Female_results_x, Female_results_y] = cyclist_gender_split(x_data, y_data, Gender);

if male_results_only == 1

clear x_data; clear y_data;

x_data = Male_results_x;

y_data = Male_results_y;

elseif female_results_only == 1

clear x_data; clear y_data;

x_data = Female_results_x;

y_data = Female_results_y;

else

end

x_max = max(x_data);

x_min = min(x_data);

y_max = max(y_data);

y_min = min(y_data);

x_mean = mean(x_data);

y_mean = mean(y_data);

x_range = linspace(0, x_max, 10000)';

%% Set up analysis and limits here!

% Equation form selector 1 = exponential, 2 = parabolic, 3 = linear, 4 = power

eq_sel = 3;

% SET UP GRID SEARCH RANGES (alin = slope of line; size = increments over alin range

%-----------------------------------------------------------------------------------

% Finer grid search --> better R^2 and fit, but over a wide alin range it is long,

% lower this but then narrow alin range below

% Set grid search size to at least 100 (or 200) * (alin lower - alin upper)

% to ensure refinement is good enough.

grid_search_size = 400;

alin_lower_lim = 0.5;

alin_upper_lim = 6;

% Setting grid search range with alin above

alin_range = linspace(alin_lower_lim, alin_upper_lim, grid_search_size)';

%% Finding minimum distances or means

% Regression with minimum total distance (in x and y) to curve from data points

% Geometric mean deviation to find min parameters

% Finds minimum (x,y) distance to a line as you iterate through alin slopes

if eq_sel == 3 % Linear

[min_alin] = min_dist_parameters_lin(alin_range, x_data, y_data, x_range);

end

%% Total least squares for given equation, once minimum is found

% dist_squared = zeros(length(a1_range),length(a2_range));

min_distances = zeros(length(x_data),1);

distances_y = zeros(length(x_data),1);

for j = 1:length(x_data)

% Check if data point is above or below curve specified by coefficients

dv1 = abs(x_range-x_data(j));

x_index = find(min(dv1) == dv1,1);

x_value_compare = x_range(x_index);

clear dv1;

% Given coefficients, find y_range and Y comparison value

if eq_sel == 3 % Linear

y_range = min_alin*x_range;

y_value_compare = min_alin*x_value_compare;

end

% Find y value closest to data point and associated x value

if y_data(j) < max(y_range)

dv1 = abs(y_range-y_data(j));

y_index = find(min(dv1) == dv1,1);

y_value_compare2 = y_range(y_index);

x_value_compare2 = x_range(y_index);

clear dv1;

else

y_value_compare2 = max(y_range);

y_index = find(max(y_range) == y_range,1);

x_value_compare2 = x_range(y_index);

end

% Set values the right way around

if x_value_compare > x_value_compare2

a = x_value_compare2;

x_value_compare2 = x_value_compare;

x_value_compare = a;

clear a;

a = y_value_compare2;

y_value_compare2 = y_value_compare;

y_value_compare = a;

clear a;

end

x_range_iter = linspace(x_value_compare, x_value_compare2, 100)';

if eq_sel == 3 % Linear

y_range_iter = min_alin*x_range_iter;

end

distances = zeros(length(x_range_iter),1);

for i = 1:length(x_range_iter)

distances(i,1) = sqrt((x_range_iter(i)-x_data(j)).^2+(y_range_iter(i)-y_data(j)).^2);

end

% Min distance for each point, and corresponding point on curve

min_distances(j,1) = min(distances);

min_distance_point_index(j,1) = find(min(distances) == distances, 1);

min_distance_point(j,1) = x_range_iter(min_distance_point_index(j,1));

min_distance_point(j,2) = y_range_iter(min_distance_point_index(j,1));

distances_y(j,1) = abs(y_value_compare-y_data(j));

end

% Funky R-squared calculation from results for total least squares regression

diff = [];

diff_y = [];

ss_min_distances = sum(min_distances.^2);

ss_regression_y = sum(distances_y.^2);

ss_total = 0;

ss_total_y = 0;

for i = 1:length(y_data)

diff(i,1) = sqrt( (y_data(i)-y_mean)^2 + (x_data(i)-x_mean)^2 );

diff_y(i,1) = y_data(i)-y_mean;

ss_total = ss_total + diff(i,1)^2;

ss_total_y = ss_total_y + diff_y(i,1)^2;

end

ss_min_distances/ss_total;

% ss_min_distances/ss_total2

R_squared = 1-(ss_min_distances/ss_total)

R_squared_y = 1-(ss_regression_y/ss_total_y)

%% Final plotting

% For plotting

alin1 = min_alin;

ylin1 = alin1*x_range;

figure

hold on

plot(x_data,y_data, 'rx')

% plot(Male_results_x(:,1),Male_results_y(:,1), 'gx')

% plot(Female_results_x(:,1),Female_results_y(:,1), 'go')

plot(x_range, ylin1, 'r')

% legend( 'Females', 'Total Least Squares Exponential Fit', 'Total Least Squares Linear Fit')

xlim([0 2500]);

ylim([0 2500]);

xlabel(x_data_label)

ylabel(y_data_label)

str = sprintf('R^2 = %1.2f', R_squared);

annotation('textbox', 'String',str,'FitBoxToText','on');

grid on

%% Results splitting functions

% function [Male_results_x, Male_results_y, Female_results_x, Female_results_y] = cyclist_gender_split(x_data, y_data, Gender)

% Male_results_x = [];

% Male_results_y = [];

% Female_results_x = [];

% Female_results_y = [];

% for i = 1:length(x_data)

% if Gender(i,1) == "Male"

% Male_results_x(end+1,1) = x_data(i,1);

% Male_results_y(end+1,1) = y_data(i,1);

% else

% Female_results_x(end+1,1) = x_data(i,1);

% Female_results_y(end+1,1) = y_data(i,1);

% end

% end

% end
